# Association between light exposure and metabolic syndrome in a rural Brazilian town

**DOI:** 10.1371/journal.pone.0238772

**Published:** 2020-09-18

**Authors:** Ana Amélia Benedito-Silva, Simon Evans, Juliana Viana Mendes, Juliana Castro, Bruno da Silva B. Gonçalves, Francieli S. Ruiz, Felipe Beijamini, Fabiana S. Evangelista, Homero Vallada, Jose Eduardo Krieger, Malcolm von Schantz, Alexandre C. Pereira, Mario Pedrazzoli

**Affiliations:** 1 School of Arts, Science and Humanities, University of São Paulo, São Paulo, Brazil; 2 Faculty of Health and Medical Sciences, University of Surrey, Guildford, Surrey, United Kingdom; 3 Institute of Psychiatry, University of São Paulo Medical School, São Paulo, Brazil; 4 Federal University of Fronteira Sul (UFFS), Realeza, Brazil; 5 Laboratory of Genetics and Molecular Cardiology, Heart Institute (InCor), University of São Paulo Medical School, São Paulo, Brazil; University of Lübeck, GERMANY

## Abstract

**Context:**

Metabolic syndrome (MetS) is a complex condition comprising a ‘clustering’ of components representing cardiometabolic risk factors for heart disease and diabetes; its prevalence rate is high and consequences serious. Evidence suggests that light exposure patterns and misalignment of circadian rhythms might contribute to MetS etiology by impacting energy metabolism and glucose regulation.

**Objective:**

We hypothesised that individuals with MetS would show disrupted circadian and sleep parameters alongside differences in light exposure profiles. We investigated this using data from a cohort study in Brazil.

**Methods:**

Data from 103 individuals from the Baependi Heart Cohort Study aged between 50 and 70 were analysed. Motor activity and light exposure were measured using wrist-worn actigraphy devices. Cardiometabolic data were used to calculate the number of MetS components present in each participant, and participants grouped as MetS/non-MetS according to standard guidelines. Between-group comparisons were made for the actigraphy measures; additionally, correlation analyses were conducted.

**Results:**

Motor activity and circadian profiles showed no differences between groups. However, the MetS group presented lower light exposure during the day and higher light exposure at night. Correlation analyses, including all participants, showed that greater daytime light exposure and greater light exposure difference between day and night were associated with reduced MetS risk (a lower number of MetS components). Also, the light exposure difference between day and night correlated with body mass index across all participants.

**Conclusions:**

The observed results suggest a direct association between light exposure and MetS which appears to not be attributable to disruptions in circadian activity rhythm nor to sleep parameters. This link between light exposure patterns and MetS risk could inform possible prevention strategies.

## Introduction

Metabolic syndrome (MetS) is a complex condition defined by a clustering of cardiometabolic components including hyperinsulinemia, glucose intolerance, dyslipidemia, obesity, and high blood pressure. Meeting criteria on at least three of these is required for a MetS diagnosis. The prevalence of MetS is increasing globally; it is estimated that 20 to 25% of the total adult population meet criteria for MetS [1–3]. The consequences are severe: there is a 1.5- to 2.5-fold increase in risk of cardiovascular events and mortality in those with MetS [3]. Consequently, MetS places substantial burden on societies and health care systems worldwide [4–7].

Insulin resistance associated with central obesity is typically regarded as the primary cause of MetS [8], driven by an imbalance between total calorie intake and total energy expenditure. Genetic factors clearly contribute to obesity risk [9]; however, the pathogenesis of MetS includes other factors such as lifestyle and metabolic consequences of obesity [8]. Physical inactivity is thought to have a role [10], and sleep quality [11] and duration [12] have also been implicated. Recent work has focused on the potential role of circadian misalignment in metabolic syndrome. Misalignment of sleep to the biological night has been shown to have a range of adverse consequences, raising risk for cardiovascular disease, diabetes, obesity, and other disorders [13]. Circadian organisation of endocrine function regulates energy homeostasis, imposing a temporal structure on various metabolic processes. Since abnormal circadian rhythms have been associated with nearly all the individual components of MetS, this has prompted some authors to suggest that circadian rhythm disturbances could be central to MetS aetiology, even proposing that MetS be renamed the ‘Circadian Syndrome’ [14]. Likely underlying mechanisms include mismatched timing of food intake, changes in gut microbiome, and dysregulation of metabolic hormones [15]. Amongst these, melatonin (a neurohormone with a diverse array of functions) plays a prominent role.

The circadian rhythm of melatonin secretion can have effects on sleep-wake cycle, as well as on the entrainment of peripheral circadian clocks [15], and has direct effects on energy metabolism via peripheral targets. Melatonin improves glucose homeostasis by protecting the pancreas against glucotoxicity [16] and exerts effects on body weight, insulin sensitivity and glucose tolerance [17,18]. Melatonin is secreted mainly during the biological night [19] and its synthesis is blocked by light via the retinal hypothalamic tract [20,21]. Contemporary lifestyles, particularly in urban communities, increase exposure to artificial light at night [22–24] which affects melatonin secretion profiles [25,26] with consequent effects on energy metabolism; the short-wavelength light produced by LED screens is particularly disruptive [15]. This could explain why large epidemiological studies have shown a direct relationship between obesity and light exposure at night, even when adjusting for other factors such as sleep duration and physical activity [27]. Studies comparing satellite images of night time illumination with obesity rates have shown that light at night correlates with obesity rate both between [28] and within [29] countries. This parallels rodent work showing that even low levels of night time light alter the timing of food intake and leads to excess weight gain [30].

It is therefore unsurprising that artificial light at night also raises MetS risk. This has been demonstrated in studies of shift workers: those who work and are exposed to high levels of light at night have a higher prevalence of MetS compared to those who only work during the day [31,32]. Simulated night shift conditions in the laboratory cause leptin reduction, insulin resistance, reversal of the rate of cortisol release and increases in blood pressure after just 10 days [33]. However, while various studies have investigated the link between MetS and shift work patterns (either in shift workers or under simulated conditions) [34], much less work has been done in community-based samples. A study in Finland found that seasonal variations in weight and appetite, as well as artificial lighting at home, strongly contributed to MetS risk [35]. Thus, light exposure patterns might be a critical (and correctable) factor in the etiology of MetS. A breakdown in circadian rhythm stability could play a causal role since irregular 24-hour activity rhythms raises risk of MetS [36]. Effects on sleep duration might also contribute since short sleep duration also raises risk of MetS [37].

To explore these possibilities, we analysed data from the Baependi Heart Study, which began in 2005 as a longitudinal cohort study to evaluate environmental and genetic effects on cardiovascular disease risk factors [38]. Baependi is a small rural town in the state of Minas Gerais, Brazil, with a traditional lifestyle and moderate and ongoing urbanization. Previous work in this cohort has shown that individuals in the rural zone show a shift of diurnal preference towards morningness [39], compared to those in the urban zone (who themselves are strongly shifted towards morningness compared to metropolitan areas). Here, we have focused on the link between MetS and patterns of light exposure in this community-dwelling sample, utilizing actigraphy and cardiometabolic data. We assessed the number of components each participant met for MetS criteria and categorized participants into either MetS or non-MetS groups accordingly. Measures of circadian rhythm stability and sleep duration were derived from motor activity collected using wrist-worn actigraphy devices. Levels of diurnal and nocturnal light exposure were also collected using the same devices. We then compared circadian rhythm and light exposure profiles between groups. Based on previous findings, we hypothesised that the MetS group would show a disruption in circadian rhythms stability and greater night-time light exposure. Follow-up correlation analyses across all participants investigated possible association between these parameters and the number of MetS components present in everyone. Finally, we investigated possible correlations between these parameters and measures of glycated haemoglobin A1c (HbA1c) and body mass index (BMI), since the evidence outlined above suggests that circadian disruption and light exposure patterns might specifically affect glucose regulation and obesity levels.

## Methods

### Participants

Participants were drawn from the Baependi Heart Cohort Study [38]. In short, the full study sample consists of 2,239 participants aged between 18 and 91 (42.5±17.2 years old). A subsample of 103 participants aged between 50 and 70 years old was invited to provide actigraphy data, since metabolic syndrome is highly age dependent [40]. These participants were distributed across 42 extended pedigrees, which from now on are referred to as “families” [41]. Included in the study were 22 families with only one participant, and 20 with more than one participant. Inclusion criteria were at least five days of complete actigraphy data and all requisite cardiometabolic parameters available.

### Ethics statement

All participants gave written informed consent before participation. This study protocol conformed to international ethics standards based on the Declaration of Helsinki and was approved by the local ethics committee (Hospital das Clínicas–Universidade de São Paulo, Brazil) (approval number 0494/10).

### Clinical parameters

The I Brazilian Guideline on Diagnosis and Treatment of Metabolic Syndrome (I-DBSM) [42] was used to categorise the non-metabolic syndrome group (non-MetS; individuals meeting criteria on less than 3 components) and the metabolic syndrome group (MetS; individuals meeting criteria on 3 or more components). The I-DBSM is based on the National Cholesterol Education Program’s Adult Treatment Panel III (NCEP-ATP III) guidelines which defines MetS criteria and components as follows: Abdominal obesity (abdominal circumference: men >102 cm, women >88 cm), increased triglycerides (≥150 mg/dL for men or women, or use of lipid-lowering medication), reduced HDL cholesterol (men <40 mg/dL, women <50 mg/dL), increased blood pressure (systolic ≥130 mmHg or diastolic ≥85 mmHg for men or women, or use of antihypertensive medication), and increased fasting glycemia (≥110 mg/dL for men or women, or presence of type 2 diabetes or diabetic treatment).

Smoking status (current, former, never) and level of education (in years) were recorded. Height and waist circumference were measured in centimetres and weight in kilograms using a calibrated digital balance. Body mass index (BMI) was calculated as body weight (kg) divided by height squared (m^2^). Systolic (SBP) and diastolic blood pressure (DBP) were calculated by taking the mean of three readings (minimum interval of 3 min between readings) using a standard digital sphygmomanometer (OMRON, Kyoto, Japan) on the left arm after 5 min rest, in the sitting position. High- and low-density lipoproteins, triglycerides, total cholesterol and fasting glucose were evaluated by standard techniques in 12-h fasting blood samples. HbA1c levels were determined by high-performance liquid chromatography (HPLC).

### Actigraphy

Participants were instructed to wear the actigraphy device that contained an accelerometer and light and temperature sensors (ActTrust AT0503, Condor Instruments, São Paulo, SP, Brazil). We analysed five consecutive days of use on their non-dominant wrist. Actigraphy measures were first calculated for each day, and then averaged. Most of the data were measured over the work week, but in a few cases, weekend data had to be included due to an excess of off-wrist occurrences. The devices recorded activity using 60-second epochs. The actigraphy software, ActStudio, provided an estimation of the sleep duration, bedtime and wake-up time based on the Kole-Kripke modified algorithm (Condor Instruments, São Paulo, Brazil). Table 1 shows motor activity and light exposure variables derived from actigraphy.

**Table 1:** Motor activity and light expositions variables obtained from actigraphy.

M10: mean diurnal motor activity (counts/min)	daily average of the 10 most active consecutive hours over 5 days [43–45]
L5: mean nocturnal motor activity (counts/min)	daily average of the 5 less active consecutive hours over 5 days [43–45]
RA: motor activity (day minus night, normalised)	RA = (M10-L5)/(M10+L5). RA values range from 0 to 1, and higher values indicate higher activity during the day relative tonight [45]
P10: phase of M10 onset (hh:min)	corresponds to the onset of the M10 phase, i.e., the onset of the 10 most active consecutive hours of motor activity
P5: phase of L5 onset (hh:min)	corresponds to the onset of L5 phase, i.e., the onset of the 5 less active consecutive hours of motor activity
IS: interdaily stability	corresponds to the circadian rhythm stability of motor activity over 5 days; a high IS value indicates a more synchronized rest/activity rhythm [46]
IV: intradaily variability	corresponds to the circadian rhythm fragmentation of motor activity; a high IV value indicates a more fragmented rest/activity rhythm (for example, occurrence of daytime naps and/or episodes of night time activity) [46]
M16_light: mean diurnal light exposure (lux)	daily average of the 16 consecutive hours with more exposure to light over 5 days
L8_light: mean nocturnal light exposure (lux)	daily average of the 8 consecutive hours with less light exposure over 5 days
RA_light: light exposure (day minus night, normalised)	RA_light = (M16_light—L8_light)/(M16_light + L8_light)). RA_light values range from 0 to 1, and higher values indicate higher light exposure during the day relative to light exposure at night [45]

### Statistical analyses

All statistical analyses were performed using the Statistical Package for the Social Sciences, version 21.0 (SPSS, Chicago, IL). Normal distribution was confirmed by the Kolmogorov–Smirnov test for all variables of interest. Data were log10 transformed prior to statistical analyses in the case of non-normal distribution (L5, IS, IV, L8_light, and RA_light, bedtime, and wake-up time). Between-group comparisons on participant characteristics were conducted using Student’s t-tests (for continuous variables) and Pearson’s χ^2^ test (for categorical variables).

A linear mixed model was used for comparisons of motor activity, light exposure, and sleep parameters, considering the family cluster as a random factor and the residential zone (urban and rural), MetS risk (MetS and non-MetS), age and sex as fixed factors.

Follow-up analyses explored Pearson's correlations between specific actigraphy parameters with a number of MetS components, with BMI and with HbA1c. The entire sample (MetS and non-MetS groups) was used for these analyses to explore possible associations in the general population. In order to avoid type I-error, Bonferroni-corrected multiple comparisons thresholds were employed. The significance level adopted in all analyses was p<0.05.

## Results

### Participant characteristics

Participant characteristics are shown in Table 2. The sample (total N = 103) was subdivided into 2 groups according to MetS diagnosis. The non-MetS group (50.5%) comprised 52 individuals and the MetS group (49.5%) comprised 51 individuals. These groups did not differ with respect to age, education, smoking status, and residential zone. The BMI was higher in the MetS group as compared to the non-MetS group (29.6±4.8 kg/m^2^ vs 24.9±4.1 kg/m^2^, p<0.001).

**Table 2:** Participant characteristics, for total and separated by MetS groups.

	Total	Non-Metabolic Syndrome	Metabolic Syndrome	p-value
N (%)	103 (100)	52 (50.5)	51 (49.5)	
Male:Female	39:64	24:28	15:36	0.080*
Age, y.o.	58.1 (5.7)	58.2 (6.0)	58.0 (5.4)	0.892^+^
Education, years	6.6 (3.8)	6.7 (5.2)	6.5 (4.3)	0.899^+^
Smoking status				0.213*
Former smokers, N (%)	36 (34.9)	17 (32.7)	19 (37.2)	
Current smokers, N (%)	15 (14.5)	5 (9.6)	10 (19.6)	
Never smokers, N (%)	49 (47.6)	30 (57.7)	19 (37.2)	
Residential zone				0.974*
Rural, N (%)	10 (9.7)	5 (9.6)	5 (9.8)	
Urban, N (%)	93 (90.3)	47 (90.4)	46 (90.2)	
BMI, kg/m2	27.2 (5.0)	24.9 (4.1)	29.6 (4.8)	***<0*.*001***^+^
High blood pressure, N (%)	38 (36.9)	14 (26.9)	24 (47.1)	*0*.034*
High waist circumference, N (%)	53 (51.5)	13 (25.0)	40 (78.4)	*<0*.0001*
Low HDL cholesterol, N (%)	60 (58.3)	17 (32.7)	43 (84.3)	*<0*.0001*
High triglycerides, N (%)	43 (41.7)	8 (15.4)	35 (68.6)	*<0*.0001*
High fasting glucose, N (%)	12 (11.7)	0 (0.0)	12 (23.5)	*<0*.0001*
Drug treatment for hypertension, N (%)	42 (40.1)	11 (21.2)	31 (60.8)	*<0*.0001*
Drug treatment for dyslipidemia, N (%)	18 (17.5)	7 (38.9)	11 (61.1)	*<0*.0001*
Drug treatment for Type 2 diabetes, N (%)	11 (10.7)	1 (1.9)	10 (19.6)	*<0*.0001*

Data are expressed as means (SD) or percentages (total N = 103) or (N = 52 or N = 51, for non-MetS or MetS groups, respectively). Significant p-values (p) are represented in **bold** and *italic*.

y.o.: Years old; HDL: High-density lipoprotein; BMI: Body mass index.

^+^Student's t-test

*Pearson's χ^2^ test.

### Light exposure, motor activity, and sleep parameters separated by groups

**Fig 1:**
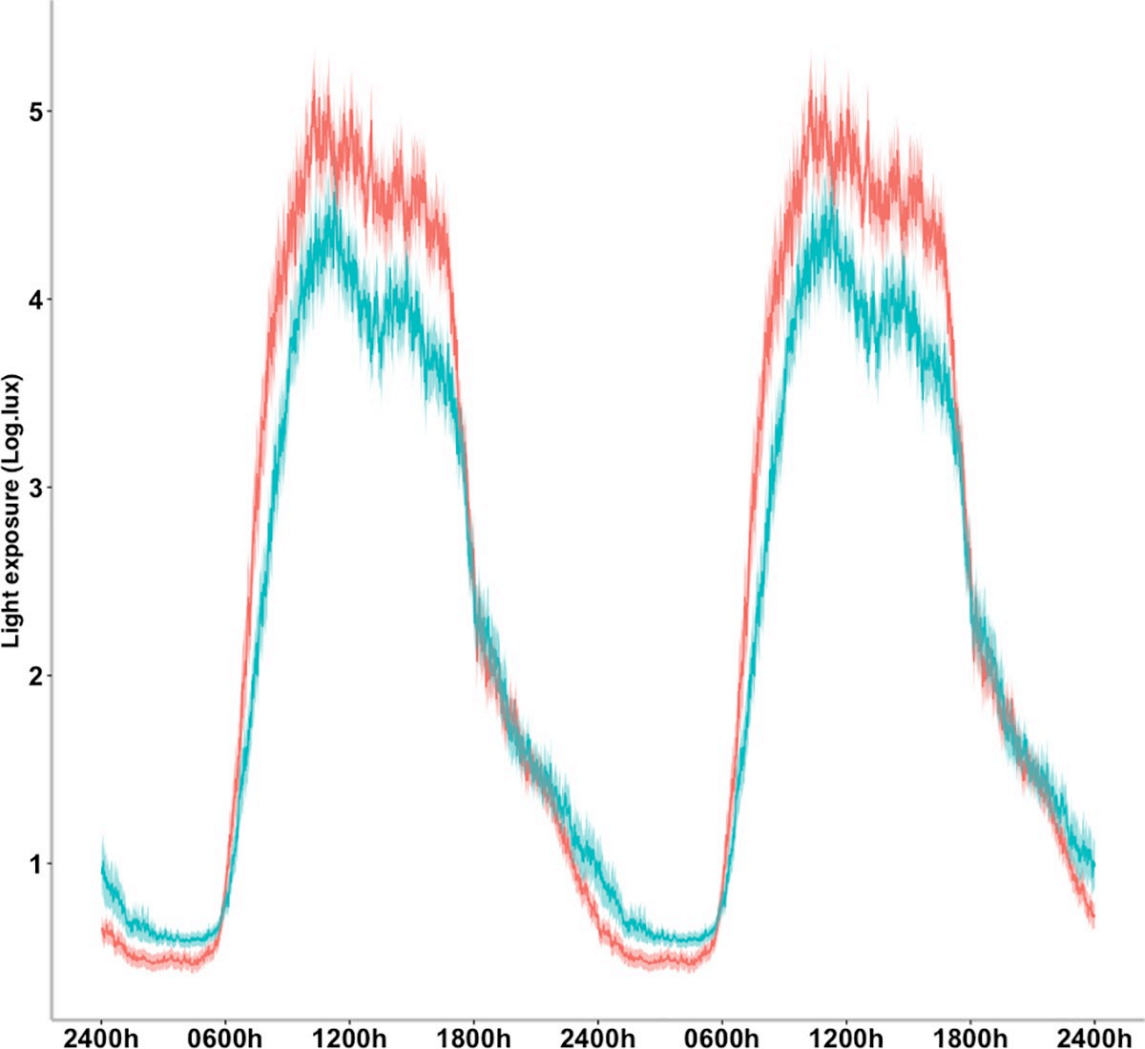
24-hour profiles of light exposure, separated by groups. Data are represented in a double plot averaged across five days. The means (bold line) and standard error of the means (shadow) are presented for the MetS group (blue) and Non-MetS group (red).

The linear mixed model analysis revealed a MetS risk group effect on light exposure parameters. The MetS group showed a decrease in diurnal light exposure (M16_light, p = 0.007) and increased in nocturnal light exposure (L8_light, p = 0.021) compared to the non-MetS group. Accordingly, the normalised difference between diurnal and nocturnal light exposure (RA_light, p = 0.016) was significantly lower in the MetS group, see Table 3. Averaged light-exposure profiles for each group are shown in Fig 1.

**Table 3:** Motor activity, light exposure and sleep variables, separated by groups.

Dependent variables	Non-Metabolic Syndrome			Metabolic Syndrome			p-value (multilevel modelling analyses)
	mean (SD)	-95% CI	+95% CI	mean (SD)	-95% CI	+95% CI	
Diurnal motor activity, M10 (counts*10^6^/min)	39.2 (13.8)	23.0	52.5	39.3 (11.8)	25.1	54.4	0.250
Nocturnal motor activity, L5 (counts*10^3^/min)	42.5 (20.2)	33.0	50.1	45.3 (22.8)	37.4	54.3	0.472
Motor activity (day minus night, normalised), RA	0.98 (0.11)	0.95	0.99	0.98 (0.12)	0.95	0.99	0.578
Diurnal motor activity onset time (min), P10	7:58 (1:30)	4:04	12:55	8:34 (2:03)	4:08	12:54	0.905
Nocturnal motor activity onset time (min), P5	01:08 (1:19)	23:08	03:42	01:32 (1:53)	23:07	3:00	0.927
Interdaily Stability, (IS)	0.59 (0.10)	0.45	0.69	0.63 (0.09)	0.49	0.73	0.090
Intradaily Variability (IV)	0.63 (0.15)	-2.3	3.5	0.62 (0.14)	-2.3	3.5	0.540
Diurnal light exposure, M16_light (lux)	3372 (784)	-374	6916	2931 (680)	-343	6341	***0*.*007***
Nocturnal light exposure, L8_light (lux)	250 (151)	102	345	305 (114)	161	404	***0*.*021***
Light exposure (day minus night,normalised), RA_ light	0.84 (0.10)	0.26	1.46	0.79 (0.09)	0.21	1.41	***0*.*016***
Sleep duration (hours:minutes)	07:09 (0:55)	03:07	11:04	7:28 (0:54)	03:02	11:38	0.238
Bedtime (hours:minutes)	22:28 (1:45)	21:01	01:32	23:05 (1:59)	20:46	01:14	0.909
Wake-up time (hours:minutes)	06:39 (1:35)	05:15	08:07	6:13 (3:58)	04:50	07:55	0.556

Multilevel modelling analysis, considering the family cluster as a random factor and metabolic syndrome status, age, address, and sex as fixed factors. Significant p-values (p) are represented in **bold** and *italic*.

SD: Standard deviation; -95% CI and +95% CI: 95% confidence interval limits.

**Fig 2:**
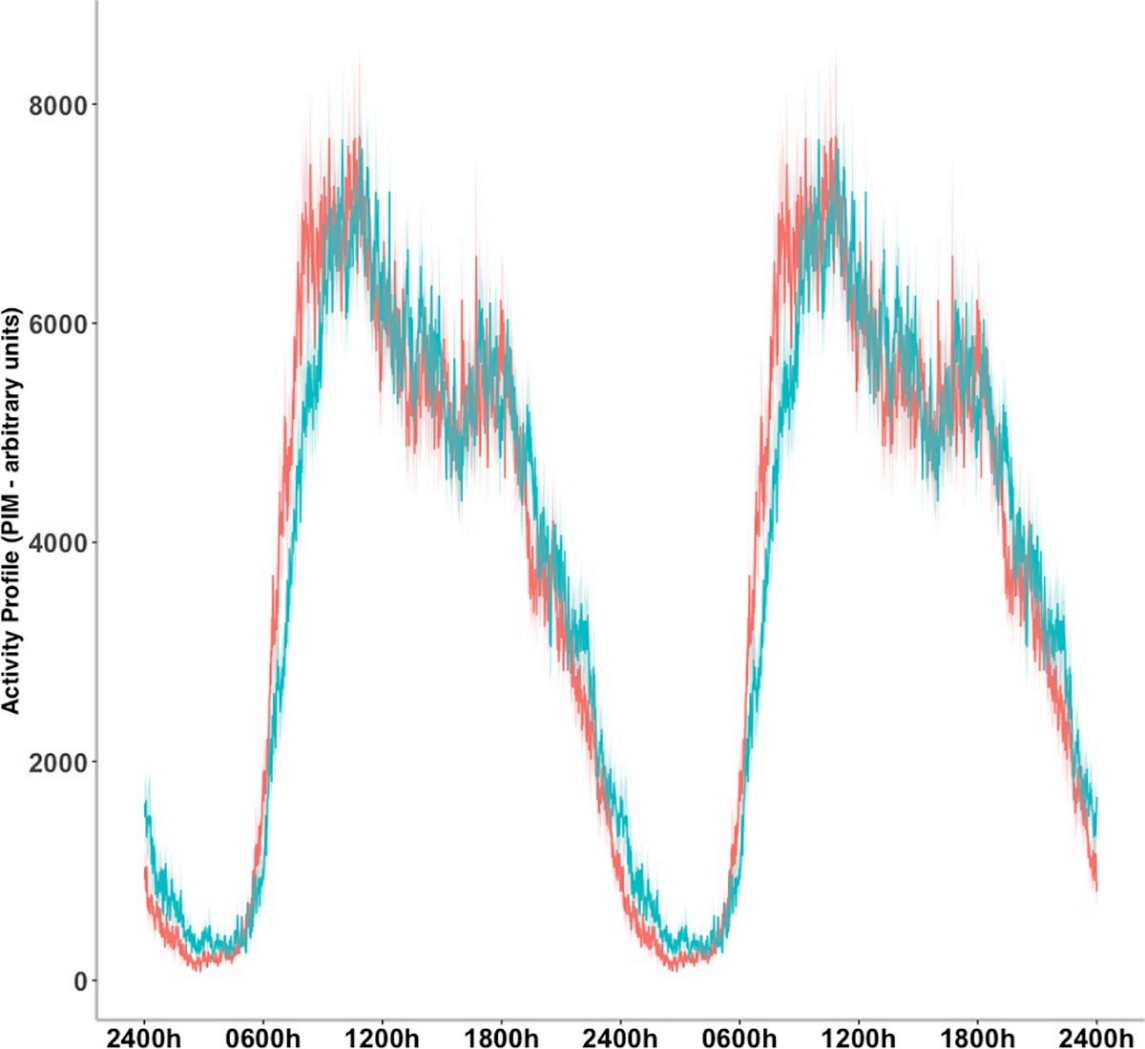
24-hour profiles of motor activity profiles, separated by groups. Data are represented in a double plot averaged across five days. The means (bold line) and standard error of the means (shadow) are presented for the MetS group (blue) and Non-MetS group (red).

There were no statistical differences between groups in the circadian parameters, interdaily stability (IS) and intradaily variability (IV). No statistical differences were observed between groups for motor activity parameters. Moreover, the averaged onset of diurnal activity (P10) occurred between 7:57 and 8:31, and the averaged onset of nocturnal activity (P5), between 24:31 and 24:58 in Non-MetS and MetS group, respectively. Averaged motor activity profiles for each group are shown in Fig 2.

Non-MetS and MetS groups did not differ in sleep parameters: bedtimes (around 23:00), wake-up times (around 06:00) and sleep durations (around 7 hours) (Table 3).

### Correlation analyses

To further explore these findings, we tested across the entire sample of participants for correlations between the number of MetS components each participant met criteria for, and the diurnal motor activity (M10), interdaily stability (IS), diurnal light exposure (M16_light), nocturnal light exposure (L8_light) and the normalised difference between diurnal and nocturnal light exposure (RA_light) parameters. Since five correlations were computed, a Bonferroni-corrected multiple comparisons threshold of 0.05/5 = 0.01 was employed. There was no correlation between number of MetS components and diurnal motor activity (M10) (r = -0.40, p = 0.692), interdaily stability (IS) (r = 0.205, p = 0.037) or nocturnal light exposure (L8_light) (r = 0.198, p = 0.045). However, there were significant negative correlations between number of MetS components and diurnal light exposure (M16_light) (r = -0.333, p = 0.001), and between number of MetS components and normalised difference between diurnal and nocturnal light exposure (RA_light) (r = -0.264, p = 0.007). Greater daytime light exposure, and a larger normalised difference between diurnal and nocturnal light exposure (RA_light) were significantly correlated with a reduced MetS risk.

**Fig 3:**
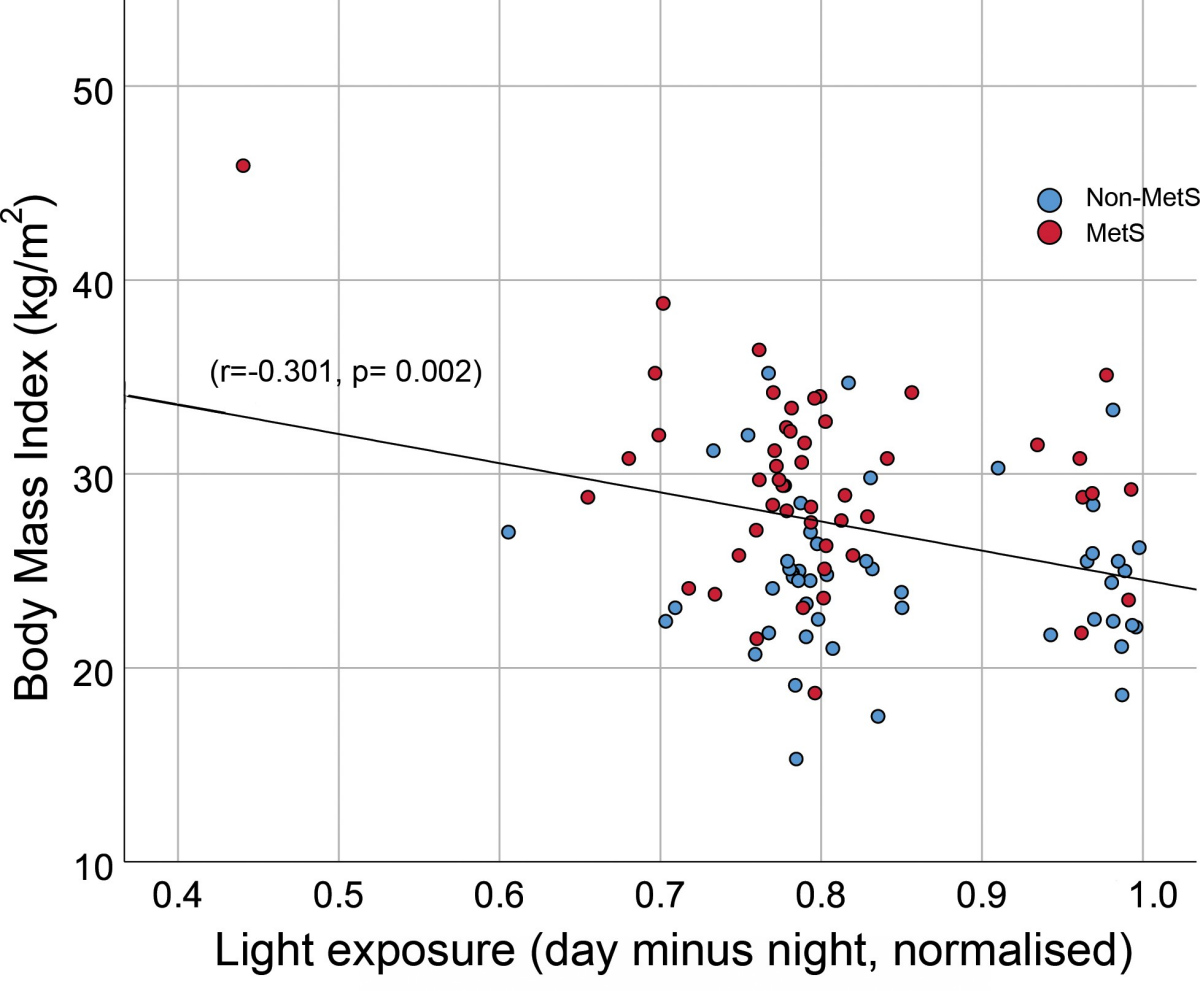
Scatterplot of BMI (body mass index) and normalised difference between diurnal and nocturnal light exposure. Data are presented with points for the MetS group (blue) and Non-MetS group (red). Across the entire sample, a line has been drawn to facilitate the calculated correlation display.

Further correlations were computed to explore effects on specific cardiometabolic parameters. Based on prior evidence we focused on glycaemic control and obesity measures (HbA1c and BMI). Correlations between these and diurnal motor activity (M10), diurnal light exposure (M16_light), nocturnal light exposure (L8_light) with the normalised difference between diurnal and nocturnal light exposure (RA_light) were assessed. A total of 8 correlations were computed requiring a Bonferroni-corrected threshold of 0.05/8 = 0.00625. The only significant correlation found was between BMI and the normalised difference between diurnal and nocturnal light exposure (RA_light) (r = -0.301, p = 0.002), see Fig 3. Evidence of other relationships were found but did not meet the Bonferroni-corrected threshold for significance: between diurnal light exposure (M16_light) and HbA1c (r = -0.210, p = 0.033), diurnal light exposure (M16_light) and BMI (r = -0.228, p = 0.023), and between diurnal motor activity (M10) and HbA1c (r = -0.235, p = 0.017).

## Discussion

This study aimed to investigate alterations in circadian rhythms in individuals with metabolic syndrome in a sample from a small rural town in Brazil. We found that individuals with a MetS diagnosis recorded considerably less light exposure during the day and more light exposure at night. Motor activity and sleep parameters did not differ between groups. This points to a specific association between light exposure profiles and MetS risk, and this was demonstrated by correlation analyses. These considered all individuals in the sample and showed that MetS risk (the number of MetS components individuals’ met criteria for) correlated with light exposure patterns. Specifically, greater diurnal light exposure and a greater normalised difference between diurnal and nocturnal light exposure (RA_light) was correlated with reduced MetS risk. RA_light was also seen to negatively correlate with BMI.

These findings accord with previous results showing links between light exposure and MetS. Shift workers exposed to high levels of light at night have a higher prevalence of MetS [31,32]. Studies using satellite imagery have linked light at night with obesity levels [28,29]. A recent study analysed self-report data of bedroom light levels and found associations with risk of weight gain in women aged 35 to 74 [47]. This is important since obesity and associated insulin resistance is critical in the etiology of MetS [8]. The current study adds further insight into these relationships. Unlike most previous work, data were collected in a naturalistic community-dwelling sample and parameters were measured objectively using actigraphy. Previous studies have focused on light at night and we found that a MetS diagnosis was associated with greater nighttime light exposure. However daytime light exposure and the normalised difference between diurnal and nocturnal light exposure (RA_light) were significantly different between MetS and non-MetS groups. Correlational analyses underlined the importance of these parameters as they correlated negatively with BMI across the whole sample. Although few previous studies have addressed this question, work by Reid *et al*. [48] showed that more light exposure earlier in the day correlated with lower BMI in a young to mid-age sample. Reid *et al*. found no effects of nighttime light, although their sample of N = 54 had low levels of nighttime light exposure overall. The current study capitalises on the high levels of variance within the Baependi population to show that individuals in this cohort with higher daytime and lower nighttime light exposure have lower BMI and less chance of a MetS diagnosis.

There are various possible mechanisms which might underlie these effects. The circadian temporal system modulates physiological homeostasis via effects on endocrine and neuroendocrine systems [see 49,50], circadian misalignment has been considered as a possible link between light exposure and metabolic disorders. Shift work has been used to study the effects of circadian misalignment, evidencing higher incidence of diabetes, obesity, and cardiovascular events [51,52] and laboratory studies of simulated shift work support these findings [33]. Since sunlight is the primary temporal cue for humans, one possibility is that greater daytime light exposure (and lower nighttime light exposure) promotes better circadian alignment with a concomitant reduction in MetS risk. However, this circadian mechanism cannot explain our results, since circadian parameters did not differ between groups. This was contrary to expectations given previous findings linking irregular activity rhythms to MetS and its components [36]. Likewise, sleep timing did not differ between groups: previous results have linked short sleep duration to MetS risk [37] although long (>10 hours) sleep duration has also been implicated [12] which might have affected our ability to detect differences. Also, given the cross-sectional nature of the current study we cannot rule out the possibility that circadian instability and short sleep earlier in life might have contributed to the development of MetS in the individuals studied here. Nevertheless, the data suggest that other mechanisms directly related to light exposure might be at play.

One possibility is a light-induced modulation of metabolic processes. In humans, studies that manipulate light exposure patterns have shown that a diminished light–dark cycle undermines the diurnal rhythm of metabolism: diet-induced thermogenesis following a meal is influenced by light exposure patterns across the day and night [53]. Melatonin is implicated in this. Even low levels of light at night can disrupt melatonin secretion with adverse effects on health [54,55] and melatonin has direct effects on energy metabolism via peripheral targets. Melatonin has an important role in regulating lipid and glucose metabolism [56]; for example, melatonin influences insulin secretion via pancreatic melatonin receptors [57]. Intervention studies provide supporting evidence. One year of melatonin treatment reduced fat mass in postmenopausal women [58] while positive effects on BMI have been shown after just 12 weeks of melatonin treatment in young obese adults [59]. The anti‐inflammatory and antioxidant effects of melatonin might also contribute [60]. We found that nighttime light exposure was greater in the MetS group, while a smaller normalized difference between day and night light exposure levels was associated with higher BMI and raised MetS risk across the whole sample. Follow up work should explore whether effects on melatonin levels might underlie these relationships.

Effects on vitamin D synthesis is another possible mechanism underlying the findings reported here. Most of our vitamin D stores derive from the absorption of sunlight by the skin, and evidence points to vitamin D deficiency as a risk factor for obesity, hypertension, diabetes and, consequently, metabolic syndrome and cardiovascular disease [61–66]. Schmitt *et al*. [67] linked vitamin D deficiency to metabolic syndrome in postmenopausal women, a sample comparable to that studied here. Women with vitamin D deficiency had a higher risk of developing MetS, hypertriglyceridemia and low HDL levels [67]; other studies have shown negative effects on total cholesterol, triglycerides and insulin [65,68,69]. Nevertheless, since results from vitamin D supplementation trials have been inconclusive [70], the beneficial effects of sunlight exposure might go beyond that of enhancing vitamin D stores. Sunlight exposure has positive effects on the immune system, reducing chronic systemic inflammation and upregulating anti‐inflammatory immune processes [71]. Also, heat from the sun can itself modulate glucose metabolism since heat increases vasodilation in peripheral tissues and promotes uptake of glucose and insulin [72]. Here, we found that across the whole sample, daytime light exposure (M16_light) correlated negatively with HbA1c levels and BMI (although these did not survive multiple comparisons correction) which is in line with these previous findings. Adequate sun exposure appears to be important for cardiometabolic health.

There are therefore multiple routes by which light exposure patterns could impact MetS risk parameters. While much previous work has focused on the effects of circadian misalignment and light at night, the current findings suggest that daytime light exposure and the normalized difference between day and night light exposure levels also strongly contribute. These might be due to effects on melatonin and/or vitamin D synthesis; sunlight exposure itself has other direct effects that could be relevant, and future studies should investigate these possibilities. Limitations of the current work include the cross-sectional design, mostly female study sample (which precludes study of sex effects) and the lack of food intake data, which could help determine how light exposure impacts caloric intake and/or timing. Strengths include the use of objective actigraphic and cardiometabolic data and the use of a sample in which MetS and non-MetS groups were well matched on demographic and physical activity (diurnal motor activity (M10)) parameters.

In conclusion, these findings provide novel insight into associations between light exposure patterns and MetS risk. A MetS diagnosis was linked to less light exposure during the day and more light exposure at night without any difference in circadian parameters. Across the whole sample, more light exposure during the day and a greater normalised difference between day and night light exposure levels correlated with reduced MetS risk. Although the data do not allow causal inferences to be made, these findings do raise the possibility that relatively straightforward lifestyle modifications (ensuring adequate daytime sun exposure and reducing bedroom light levels) could be effective in reducing the prevalence of MetS. Since the current results were observed in a semi-rural environment where individuals tend to have relatively high/low levels of day/night light exposure, such effects might be even more pronounced in urban environments and merit further investigation.

## Supporting information

S1 Table Individual values of the actigraphic data of the study participants. (XLSX)
